# Global patterns and determinants of household medicine storage and disposal: a systematic review and meta-analysis

**DOI:** 10.1080/20523211.2025.2601936

**Published:** 2026-01-20

**Authors:** Guu Nandar Chit, Su Myat Thin, Anuchai Theeraroungchaisri, Suntaree Watcharadamrongkun, Tanattha Kittisopee

**Affiliations:** a Department of Social and Administrative Pharmacy, Faculty of Pharmaceutical Sciences, Chulalongkorn University, Bangkok, Thailand; b Faculty of Pharmacy, Siam University, Bangkok, Thailand

**Keywords:** Unused medicines, expired medicines, drug storage, drug disposal

## Abstract

**Background:**

Improper storage and disposal of unused and expired medicines at home have become a global challenge, creating significant hazards to public health and the environment. This systematic review aimed to explore the prevalence of proper storage and disposal of medicines and to examine factors influencing these proper behaviours.

**Method:**

We searched PubMed, Scopus, Springer Link, Science Direct, and EBSCO for articles published between 1 January 1990, and 31 May 2023. Inclusion criteria included studies conducted about medicines in a household setting, articles written in the English language, and full text that could be retrieved. Exclusion criteria included pilot studies, editorials, or opinions. The QualSyst assessment tool was applied to evaluate the quality of the included studies. This systematic review was conducted and reported in accordance with the PRISMA 2020 (Preferred Reporting Items for Systematic Reviews and Meta-Analyses) guidelines. The prevalence of proper storage and disposal, determinants of proper and improper storage, and proper disposal of medicines were elaborated using both quantitative and qualitative methods.

**Results:**

The search found 822 articles and included 61 eligible studies from 27 countries. The overall prevalence of proper medicine storage was only 45%. The proper disposal rates were only 13% for unused medicines and 8% for expired medicines. Proper storage was positively associated with knowledge, education, storage counselling, presence of chronic illness, household size, and the number of children in the household; and negatively associated with age and male gender. Proper disposal was positively associated with awareness, peer influence, knowledge, education, and having family members who are incapable of independently managing their medicine.

**Conclusion:**

Proper storage guidance should be included on a medicine label for every medicine dispensed by pharmacists. The take-back programmes campaign and public education related to proper disposal of medicine were recommended to save the environment and public health.

## Background

The global use of medicines is gradually rising (Kumari et al., 2022) and is expected to reach 4.5 trillion doses by 2020, up 24% from 2015 (Aitken & Kleinrock, 2015), and it is projected to increase 1.6% slowly through 2027 (Aitken et al., 2023). The increased usage of medicines results in an abundant stock of medicines in households (Abahussain & Ball, 2007), which can increase improper storage of medicines (Yin et al., 2019). Improper storage of medicine reduces the efficacy of medicines and increases the risks of misuse and unintentional poisoning in children or pets (Kusturica et al., 2012; U.S. Food & Drug Administration, 2013, 2024). A proper storage condition was defined as storing the medicines in a ventilated area, in a refrigerated environment when needed, and away from the reach of children (Mui et al., 2019; WHO, 2021, 1999). The storage of medicines under conditions such as temperature fluctuations, intense light, or high humidity (Mui et al., 2019; WHO, 2021, 1999; Yousif, 2002), which can shorten their shelf life, such as keeping them in a car, or in places with frequent movement, such as handbags or suitcases, where labels and packaging can be damaged, was considered improper storage.

The disposal of unused, damaged, and expired medicines becomes a global challenge regardless of their importance (Aluko et al., 2022). The incorrect disposal of medicines could be a serious environmental (Abahussain & Ball, 2007; Bound & Voulvoulis, 2005; Persson et al., 2009) and community health risk (Adedeji-Adenola et al., 2022; Maeng et al., 2016; Naser et al., 2021). The drug residues may therefore be diffused into landfill, groundwater, surface water, and even drinking water, which leads to environmental pollution (Bound & Voulvoulis, 2005; Persson et al., 2009). There is proof that the presence of antibiotics in the water affects the bacteria existing and results in antibiotic resistance as well as antimicrobial resistance (Costanzo et al., 2005; Gyesi et al., 2022; Sharma et al., 2021). Proper disposal of medicines is defined as returning to the designated authorities or place (Maldives Food and Drug Authority; Mui et al., 2019; National Medicine Regulatory Authority, 2021; NHS Frimley Clinical Commisioning Group, 2022; Pharmaceutical Society of Ireland, 2017; Queeensland Government;; U.S. Food & Drug Administration, 2013, 2024; WHO, 2021, 1999), and incineration under supervision of authority (National Medicine Regulatory Authority, 2021; WHO, 2021, 1999). Improper disposal of medicines is defined as throwing in household trash or garbage (Pharmaceutical Society of Ireland, 2017), flushing down the toilet or sink (Mui et al., 2019; National Medicine Regulatory Authority, 2021; NHS Frimley Clinical Commisioning Group, 2022; Pharmaceutical Society of Ireland, 2017; WHO, 2021, 1999), and illegal burning (Mui et al., 2019).

In order to prevent improper disposal, there is a need to find factors influencing proper and improper disposal. While numerous studies have addressed aspects of household medicine management, a comprehensive systematic review and meta-analysis focusing specifically on the global prevalence and determinants of proper storage and disposal practices is essential for developing evidence-based policy. Therefore, this study aims to review the factors influencing these behaviours systematically and also describe and explore how people manage unused and expired medicines in order to raise awareness among policymakers, which would be useful for establishing the standard protocols for the proper disposal of medicines.

## Methods

### Search sources and strategies

This systematic review was conducted and reported in accordance with the PRISMA 2020 (Preferred Reporting Items for Systematic Reviews and Meta-Analyses) guidelines. The searches were conducted through online databases (PubMed, Scopus, Springer Link, ScienceDirect, and EBSCO). To cover more data, grey literature (government websites, guidelines), which are related to medication storage and disposal, were also thoroughly searched for the cited references.

The search terms were (unused) OR (expir*) AND (medicine) OR (drug) OR (pharmaceutical) OR (medication) AND (stor*) OR (dispos*) OR (wast*) OR (destroy*) AND (house*) OR (home) OR (resident*) NOT (hospital) OR (pharmacy) (Supplemental Appendix A). The logical expression pattern was adjusted according to the database searching guideline. We conducted a search for articles published between 1 January 1990 and 31 May 2023.

### Study selection

First, the titles and abstracts were screened to meet the inclusion criteria, which were (1) studies conducted on medicines in a household setting, (2) articles written in the English language, and (3) full text that could be retrieved. Pilot studies, editorials, or opinions were excluded. The included studies were screened manually by one researcher (G.N.C.) based on the criteria for eligibility and checked by another researcher (A.T.*).

### Quality assessment of articles

The QualSyst tool developed by Kmet, Lee, and Cook was applied to evaluate the quality of included studies (Kmet et al., 2004). The assessment tool has 14 criteria for evaluating quantitative studies and 10 criteria for qualitative studies, with a rating system of 0 for not applicable, 1 for partially applicable, 2 for totally applicable, and N/A for not applicable for some studies (Supplemental Appendices B and C). The studies with a quality score of less than 0.75 were excluded. The quality assessment score was performed independently by three researchers (G.N.C., A.T.*, and S.M.T.) (Supplemental Appendices D and E).

### Data extraction

The percentages of household medicine storage, storage locations, disposal methods, factors influencing the storage, and factors influencing the disposal of medicines were extracted by two researchers (G.N.C. & A.T.). The two researchers also classified proper storage and disposal cases. According to WHO, the proper storage of medications refers to maintaining medication products under conditions that preserve their safety, stability, potency, and efficacy throughout their shelf life, in accordance with manufacturer instructions, regulatory standards, and contextual considerations. This includes temperature control at a specific temperature range for specific drugs, humidity protection away from excessive moisture, protecting medications from direct sunlight or UV exposure, and safety and security from the sight of children, unauthorised persons, or those at risk of misuse (Mui et al., 2019; WHO, 2021, 1999; Yousif, 2002). Since the definition dismisses the nuance of storage and disposal guidelines that differ by type of medication and region (e.g. country, state, province, etc.), the researcher also took into account the type of medication and study locations for the reliability and validity of the classifications.

To classify proper disposal cases, the definition of proper medicine disposal refers to the safe and secure elimination of unused or expired medicine to prevent harmful consequences to individuals, communities, and the environment. According to WHO and countries’ responsible organisation guidelines, the proper disposal includes returning to pharmacies or authorised take-back programmes, incineration under supervision of authority, and following local or national guidelines (Maldives Food and Drug Authority; Mui et al., 2019; National Medicine Regulatory Authority, 2021; NHS Frimley Clinical Commisioning Group, 2022; Pharmaceutical Society of Ireland, 2017; Queeensland Government; U.S. Food & Drug Administration, 2013, 2024; WHO, 2021, 1999).

To solve the incongruency, the two researchers discussed it with other researchers (T.K. and S.W.). The data for author name, publication year, country where the study was conducted, and sample population were also extracted.

### Data analysis

#### Quantitative systematic review

A quantitative systematic review, meta-analysis, was used to find the prevalence of proper storage and proper disposal of medicines by using STATA software, version 19.5 (Stata Corp LLC, College Station, TX, USA). To assess the heterogeneity among studies, the Cochrane Q statistic and I^2^ index were used (Deeks et al., 2019). As the Q test for heterogeneity was significant, the random-effects model was used.

The prevalence of proper storage was calculated from the number of participants who conducted proper storage actions and the total sample size. The prevalence of proper disposal was calculated from the number of participants who conducted proper disposal action and the total sample size. The results were reported along with 95% confidence intervals (95% CI). Subgroup analyses according to geographical regions were also performed to distinguish the proper storage and disposal among areas.

#### Qualitative systematic review

The storage locations, disposal methods, and factors influencing proper and improper storage and disposal methods were summarised using a qualitative systematic review. A content analysis was approached inductively, with codes originating directly from the data. Most of the terms had come from original articles. The places like car, handbag, pocket, suitcase, purse, basket, box, cupboard in garage, open location (e.g.. on the table), storeroom, and around the house were coded as ‘unsuitable place’ inthe storage location section. For the disposal methods, flushing down the toilet, sink, river or lake was coded as the ‘water drainage’.

Two researchers (G.N.C. and A.T.*) independently performed a content analysis by manual coding to categorise the information within each study and manually analysed the data to identify patterns. Two additional researchers (T.K. and S.M.T.) double-reviewed the codes and verified the analysis.

## Result

**Figure 1.**
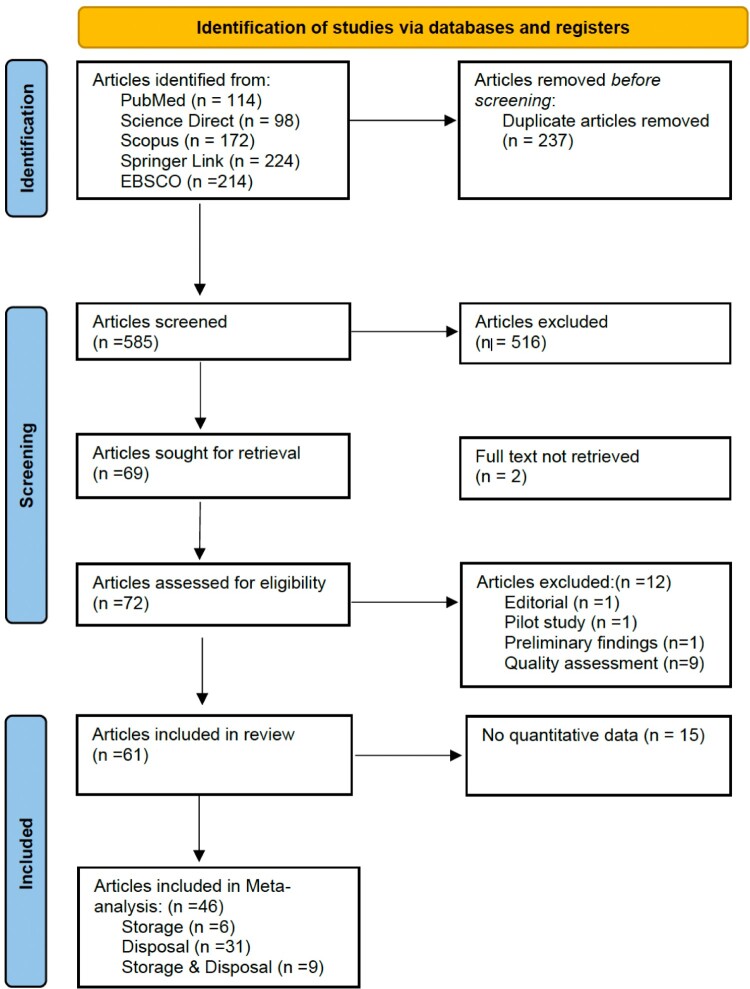
PRISMA Flow Chart of included studies in the review.

A total of 822 articles were found in five databases, and 237 articles were duplicates. The titles and abstracts of 585 articles were screened, and 516 articles were excluded because they were not related to the storage and disposal of medicines in a household setting. The remaining 72 articles were retrieved. Five articles were excluded because of the unavailability of full text (n = 2), a pilot study (n = 1), an editorial (n = 1), and preliminary findings (n = 1). After assessing the quality score, 9 articles were excluded (Supplemental Appendix F). Finally, 61 articles were included in the current systematic review (Figure 1).

### Characteristics of the included studies

This systematic review included 61 studies that surveyed 34,297 participants across 27 countries, with sample sizes ranging from 28 to 4,302. There were 25 studies conducted in Asia (Abushanab et al., 2013; Al-Azzam et al., 2012; Al-Naggar, 2010; Al-Samydai et al., 2022; Althagafi et al., 2022; Ana et al., 2023; Bashaar et al., 2017; Elghazaly et al., 2023; Hajj et al., 2022; Hassan et al., 2022; Insani et al., 2020; Khansa et al., 2023; Kumar et al., 2019; Manocha et al., 2020; Naser et al., 2021; Nisha et al., 2022; Rashid et al., 2022; Sarraf et al., 2022; Sawalha, 2010; Shoaib et al., 2022; Sweileh et al., 2010; Wajid et al., 2020; Wang et al., 2021; Yin et al., 2019; Zargarzadeh et al., 2005), 16 studies in Africa (Addis, 2023; Adedeji-Adenola et al., 2022; Akande-Sholabi et al., 2023; Aluko et al., 2022; Asmamaw et al., 2023; Kahsay et al., 2020; Kesentseng Jackson; Mahlaba et al., 2022; Mitiku et al., 2024; Nakiganda et al., 2023; Temu et al., 2006; Teni et al., 2017; Toe et al., 2023; WM-Bekele et al., 2023; Woldeyohanins et al., 2021; Yimenu et al., 2020; Yohanes & Mulu, 2018), 6 studies in Europe (Mina et al., 2019; Terzic-Supic et al., 2019; Vellinga et al., 2014; Watkins et al., 2022; West et al., 2016; Zorpas et al., 2018), 8 studies in North America (Calderón & Tarapués, 2021; Egan et al., 2019; Egan et al., 2020; Engster et al., 2019; Gregorian et al., 2020; Kennedy-Hendricks et al., 2016; Reddy et al., 2014; Stone et al., 2025), 4 studies in South America (Fernandes et al., 2020; Lenita et al., 2019; Martins et al., 2017; Rausch et al., 2023), and 2 studies in Oceania (Bettington et al., 2018; Kelly et al., 2018). In terms of the study participants, one study was conducted on healthcare professionals (Sarraf et al., 2022), two on undergraduate health professions students (Akande-Sholabi et al., 2023; Nakiganda et al., 2023), and the remaining studies were done on the general public (Abushanab et al., 2013; Addis, 2023; Adedeji-Adenola et al., 2022; Al-Azzam et al., 2012; Al-Naggar, 2010; Al-Samydai et al., 2022; Althagafi et al., 2022; Aluko et al., 2022; Ana et al., 2023; Asmamaw et al., 2023; Bashaar et al., 2017; Bettington et al., 2018; Calderón & Tarapués, 2021; Egan et al., 2019; Egan et al., 2020; Elghazaly et al., 2023; Engster et al., 2019; Fernandes et al., 2020; Gregorian et al., 2020; Hajj et al., 2022; Hassan et al., 2022; Insani et al., 2020; Kahsay et al., 2020; Kelly et al., 2018; Kennedy-Hendricks et al., 2016; Khansa et al., 2023; Kumar et al., 2019; Lenita et al., 2019; Kesentseng Jackson Mahlaba et al., 2022; Manocha et al., 2020; Martins et al., 2017; Mina et al., 2019; Mitiku et al., 2024; Naser et al., 2021; Rashid et al., 2022; Rausch et al., 2023; Reddy et al., 2014; Sawalha, 2010; Shoaib et al., 2022; Sweileh et al., 2010; Temu et al., 2006; Teni et al., 2017; Terzic-Supic et al., 2019; Toe et al., 2023; Vellinga et al., 2014; Wajid et al., 2020; Wang et al., 2021; Watkins et al., 2022; West et al., 2016; WM-Bekele et al., 2023; Woldeyohanins et al., 2021; Yimenu et al., 2020; Yin et al., 2019; Yohanes & Mulu, 2018; Zargarzadeh et al., 2005; Zorpas et al., 2018). The characteristics of included studies are shown in Table 1.

**Table 1.** Characteristics of the included studies.

Author, Year, Study place	Number of participants	Percentage of medicine storage at home	Storage Location	Disposal Methods
Elghazaly et al. (2023), Saudi Arabia	604^(b)^	–	Refrigerator Bedrooms Home pharmacies Secure locations	–
Gregorian et al. (2020), USA	500^(b)^	–	Open location Concealed location Locked location	–
Teni et al. (2017), Ethiopia	771^(b)^	–	Drawer Refrigerator Table Bag/purse Pockets on cloth	–
Abushanab et al. (2013), Jordan	219^(b)^	46%	Pharmacy cabinets Refrigerator Around the house (kitchen, bathroom, living room and bedrooms)	–
Al-Azzam et al. (2012), Jordan	435^(b)^	–	Refrigerator Kitchen Dining room Bedroom First aid box	–
Sweileh et al. (2010), Palestine	415^(b)^	–	Pharmacy cabinet Other places around the house (kitchen and bedrooms) Refrigerator	–
Sweileh et al. (2010), Palestine	415^(b)^	–	Pharmacy cabinets Refrigerator Around the house (bedrooms, bathroom and kitchen)	–
Temu et al. (2006), Tanzania	300^(b)^	22%	Special containers Cupboards Open spaces (such as on the table)	–
Mitiku et al. (2024), Ethiopia	397^(b)^	–	Shelf Cupboard Box Cabinet	–
Nakiganda et al. (2023), Uganda	205^(a)^	–	Drawer Suitcase Safety cabinet Handbag First aid box	Household garbage Burning with rubbish Flushing them in toilets/sinks Taken back medicines to pharmacies
Hajj et al. (2022), Lebanon	735^(b)^	–	Bedroom Kitchen Storage room Bathroom Refrigerator Car	Household garbage Flush in the toilet/sink Donate to charitable institutions Return it to the pharmacy Burn
Rashid et al. (2022), Malaysia	350^(b)^	–	In the kitchen In the bedroom In your handbag Inside the refrigerator In the mobile medical box	Return to MOH facilities Return to private clinic or retail pharmacy Give to friends or relatives Throw away in household garbage Flush unused medications in toilet or sink
Shoaib et al. (2022), Pakistan	830^(b)^	–	Kitchen cabinet Bathroom cabinet Bedroom cabinet Medicine box Refrigerator	The exchange at the pharmacies Throw away in dustbins (household trash) Give to hospitals/clinic Give to friends or relatives Flush in toilet or sink Return them to pharmacies or hospitals for disposal
Althagafi et al. (2022), Saudi Arabia	1105^(b)^	–	Refrigerator Bedroom Living room Locked location Medicine cabinet	Household trash bins Toilet flush Return to pharmacy Return to hospital Give/donate medications to other people
Hassan et al. (2022), Saudi Arabia	820^(b)^	–	Refrigerator Bedroom Kitchen Living room Car	Throw it in the garbage Store it for future use Give it to a friend or relative Return it to the pharmacy Throw it in the toilet Burn it Buried in the soil
Mahlaba et al. (2022), South Africa	171^(b)^	–	Cupboard Bedroom Fridge Kitchen Box/multiple storage boxes Bathroom	Flush down the toilet Flush down the basin Municipal bin Pit toilet Return to a health-care facility Burning it Bury it underground Give to friends and family
Watkins et al. (2022), UK	663^(b)^	–	Kitchen/bathroom All other storage	Bin Return to pharmacy Sink/toilet
Naser et al. (2021), Jordan	1092^(b)^	–	Bedroom Kitchen Store room Bathroom	Throw them in the garbage Rinsing down a sink Returning to a pharmacist Flushing down a toilet Giving away to friends or relatives
Fernandes et al. (2020), Brazil	423^(b)^	56.1%	Kitchen Bedroom Living/pantry room Bathroom	Drops off at primary care unit or gives back to health agent Drops off at public/private pharmacy Donates to neighbors/friends/relatives Disposes in domestic waste Disposes in toilet Disposes in kitchen/bathroom sink, rivers or lakes
Yimenu et al. (2020), Ethiopia	507^(b)^	97%	Shelf Box Pocket Bag Basket Cupboard	Burn Bury in the ground Flush down the toilet Give to ill person Return back to health facility Throw in the thrash
Mina et al. (2019), Serbia	70^(b)^	58.1%	Living room Kitchen Bathroom Pantry Bedroom	Trash Return to pharmacy Burn Give to friends/family
Terzic-Supic et al. (2019), Serbia	609^(b)^	84%	Box Drawer Living room Bedroom kitchen	Trash Return to pharmacy
Martins et al. (2017), Brazil	267^(b)^	60%	Kitchen Living room Bedroom	Home garbage or public sewage system Returned to health facilities
West et al. (2016), Malta	391^(b)^	44.2%	Medication cabinets in kitchen Medication cabinets in bedroom Medication cabinets in bathroom Medication cabinets in garage Cupboard in kitchen Cupboard in bedroom Cupboard in bathroom Cupboard in garage Office Car Fridge Carried around by individual	Household rubbish Toilet or Sink Give them to another person or friend Take them to a medication disposal bring-in-site Give them to a pharmacy
Vellinga et al. (2014), Ireland	398^(b)^	95.3%	Kitchen Bedroom Bathroom	Household waste Sink Toilet
Reddy et al. (2014), USA	300^(b)^	88%	Where everybody can see Hidden but not locked Under lock and key	Giving them to doctor/pharmacy for disposal Flushing down the toilet Throwing in the trash
Kennedy-Hendricks et al. (2016), USA	1032^(b)^	–	Location that locks Location that locks or latches	Flush down the toilet Throw out in the trash Turn in to pharmacist or ‘take-back' programme
Engster et al. (2019), USA	486^(b)^	–	Out of sight In plain sight	Trash Flushing down toilet Prescription takeback programme
Rausch et al. (2023), Brazil	156^(b)^	–	–	Public Health Clinic Household garbage Sinks or toilet
Addis (2023), Ethiopia	354^(b)^	–	–	Threw into the trash Give them to another ill person Keeping them in the house Flushing them down the toilet Threw them into the trash & flushing them down the toilet Buried them in ground Threw them into the trash & buried them in ground Threw them into the environment Burning them Returning to a nearby pharmacy
WM-Bekele et al. (2023), Ethiopia	405^(b)^	–	–	Household garbage bins Flushed down the toilet Buried Burn
Khansa et al. (2023), Lebanon	385^(b)^	–	–	Household garbage Flush in toilet/sink Return back to the pharmacy
Toe et al. (2023), Liberia	300^(b)^	–	–	Flush down in toilet Rinsing down a tank Return to pharmacy Municipality collect from home Giving to people in need
Akande-Sholabi et al. (2023), Nigeria	930^(a)^	–	–	Throw it in the trash Flush it down the toilet Return it to a pharmacy or drug take-back programme Give it to someone who needs it
Nisha et al. (2022), Nepal	210^(b)^	–	–	Household Garbage
Sarraf et al. (2022), Nepal	294^(a)^	–	–	Sink or flush down the toilet Incinerate/burn Household garbage River or lake Bury Give them back to the pharmacy Donate to hospitals or other organisations Give them to friends or relatives
Adedeji-Adenola et al. (2022), Nigeria	534^(b)^	–	–	Burn Return to pharmacy Donate to hospital Household garbage Toilet/sink Give to friends or relatives
Aluko et al. (2022), Nigeria	290^(b)^	56.2%	–	Give to other people Household garbage Pour in the toilet/sink Bury Burn
Calderón and Tarapués (2021), Ecuador	498^(b)^	–	–	Flushed down the toilet Household garbage
Woldeyohanins et al. (2021), Ethiopia	404^(b)^	95%	–	Household garbage Toilet/sink Burn Give to friends or relatives Return to pharmacy
Wang et al. (2021), Malaysia	1184^(b)^	–	–	Kept it for future use Give them to Medicine-Return-Programme (MRP) facility Trash/garbage bin Toilet/drain Gave it to someone who would use it
Kahsay et al. (2020), Ethiopia	359^(b)^	–	–	Household garbage Donating them to hospital Giving them to friends or relatives Returning them back to pharmacy Keeping them at home until expired Flushing them in toilet Burning them
Manocha et al. (2020), India	956^(b)^	77.7%	–	Flushed in sewer water Throw away in Household trash Bury in the ground Burning at home Return to the medical store
Insani et al. (2020), Indonesia	497^(b)^	92.1%	–	Threw away in household garbage Gave to friends/relatives Flushed down to the toilet or sink Donated to the hospital Burned the medicine Returned it to pharmacy
Wajid et al. (2020), Saudi Arabia	337^(b)^	86.6%	–	Throw away in the household garbage Donate to a hospital Give to friends or relatives Return to medical stores Flush unused medicines in the toilet
Lenita et al. (2019), Brazil	99^(b)^	17.9%	–	Trash Reuse Return to the place of purchase Toilet
Egan et al. (2019), USA	3043^(b)^	–	–	Trash Flush Drop-box Take-back Event
Bettington et al. (2018), Australia	4302^(b)^	–	–	Household garbage Pour down the drain or toilet Return to the pharmacy
Zorpas et al. (2018), Cyprus	184^(b)^	–	–	Garbage Toilets/sink Reuse Illegal burn
Yohanes and Mulu (2018), Ethiopia	694^(b)^	–	–	Throw away in household garbage Flush unused medications in toilet/sink Burn Donate to hospital Give to friends or relatives Return back to pharmacy
Bashaar et al. (2017), Afghanistan	301^(b)^	76.8%	–	Household garbage Donate to hospital Give to friends or relatives Return to medical stores Toilet or sink
Al-Naggar (2010), Malaysia	28^(b)^	70.1%	–	Trash Burning Refrigerator Return to hospital Buried in the ground Flush down the toilet
Egan et al. (2020), USA	627^(b)^	–	–	Takeback event Dropbox Flush Trash
Stone et al. (2025), USA	161^(b)^	–	–	Mixed with something and threw away Pharmacy Law enforcement Toilet or sink Threw away (without mixing with something)
Asmamaw et al. (2023), Ethiopia	348^(b)^	–	–	Put into latrine Throw away in household garbage A gift to friends or relatives Keep until expired Return to healthcare facilities/professionals Others(Burn, landfill)
Ana et al. (2023), Indonesia	96^(b)^	–	–	–
Al-Samydai et al. (2022), Jordan	450^(b)^	49.8%	–	–
Kumar et al. (2019), India	145^(b)^	–	–	–
Yin et al. (2019), China	625^(b)^	55.9%	–	–
Kelly et al. (2018), Australia	166^(b)^	95.5%	–	–
Zargarzadeh et al. (2005), Iran	512^(b)^	–	–	–

^Note: a^ ^=^ ^healthcare professionals, b^ ^=^ ^general public, daches indicate information unavailable.^

### Extent of households having medicine storage

**Figure 2.**
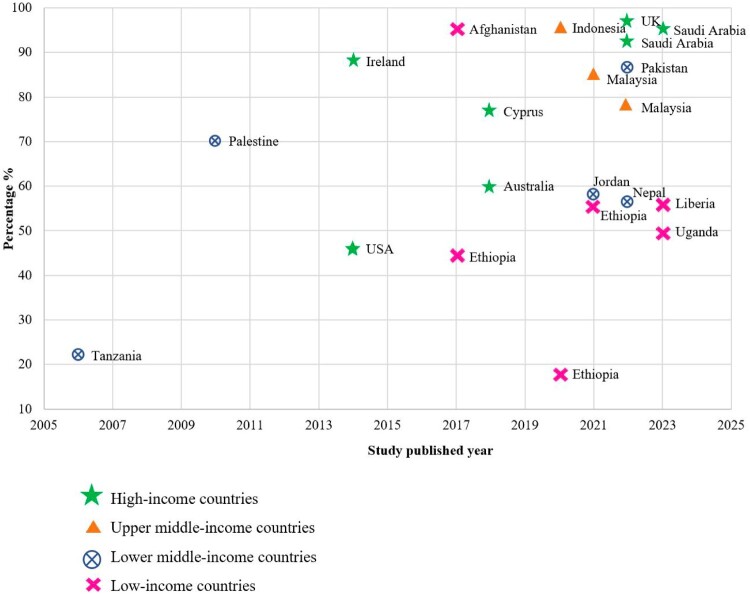
Extent of household having medicine storage.

Of the 61 studies, 21 studies reported the extent of medicine storage among households (Althagafi et al., 2022; Bashaar et al., 2017; Bettington et al., 2018; Elghazaly et al., 2023; Insani et al., 2020; Nakiganda et al., 2023; Naser et al., 2021; Rashid et al., 2022; Reddy et al., 2014; Sarraf et al., 2022; Sawalha, 2010; Shoaib et al., 2022; Temu et al., 2006; Teni et al., 2017; Toe et al., 2023; Vellinga et al., 2014; Wang et al., 2021; Watkins et al., 2022; Woldeyohanins et al., 2021; Yimenu et al., 2020; Zorpas et al., 2018). The percentage of medicine storage at home varied from 17.9% to 97% within the years 2006–2023 (Watkins et al., 2022; Yimenu et al., 2020) (Figure 2). The World Bank’s income classification system was applied to categorise countries according to their income levels: low-income, lower middle-income, upper middle-income, and high-income (The World Bank Group). Since 2021, lower-middle-income and low-income countries (ranging from 17.9% (Yimenu et al., 2020) to 95.3% (Bashaar et al., 2017)) were less likely to store medicines at home than high-income countries.

### The prevalence of proper storage

**Figure 3.**
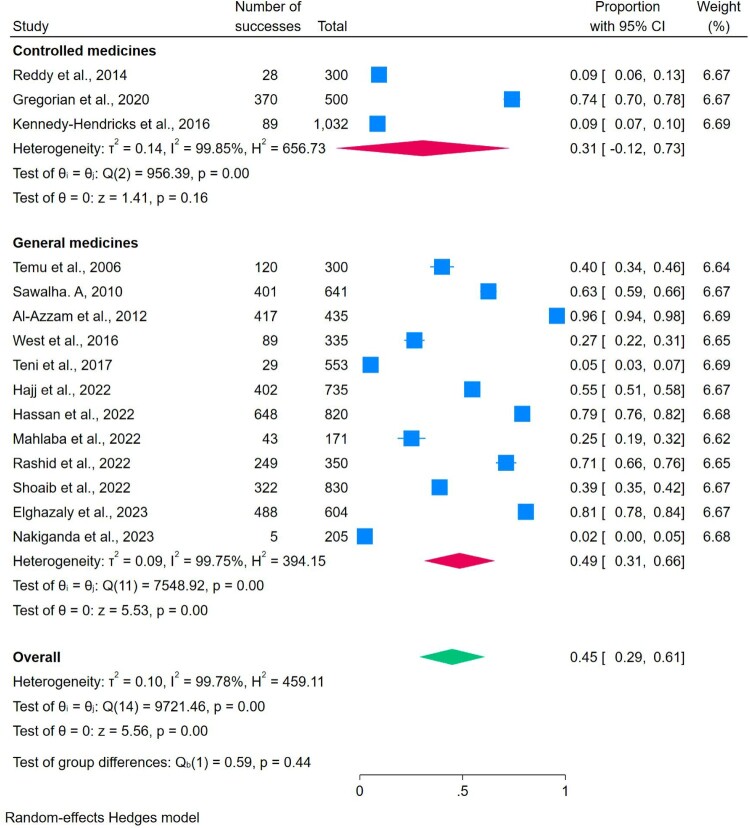
The prevalence of proper storage practice.

Among the included 61 studies, 15 studies reported proper storage locations among households (Al-Azzam et al., 2012; Elghazaly et al., 2023; Gregorian et al., 2020; Hajj et al., 2022; Hassan et al., 2022; Kennedy-Hendricks et al., 2016; K. J. Mahlaba et al., 2022; Nakiganda et al., 2023; Rashid et al., 2022; Sawalha, 2010; Temu et al., 2006; Teni et al., 2017; West et al., 2016). Storing the medicines in a ventilated area, in a refrigerated environment when needed, and away from the reach of children was considered a proper storage condition. There was significant heterogeneity among the studies (I² = 99.78%, *p* = 0.00; Q = 9721.46). Therefore, a random-effects model was applied. The overall prevalence of proper storage practices was estimated at 45% (95% CI: 20–61). The prevalence of proper storage for general medicines is 49%, and for controlled medicines is 31% (Figure 3).

#### Proper medicine storage and countries’ income levels

**Figure 4.**
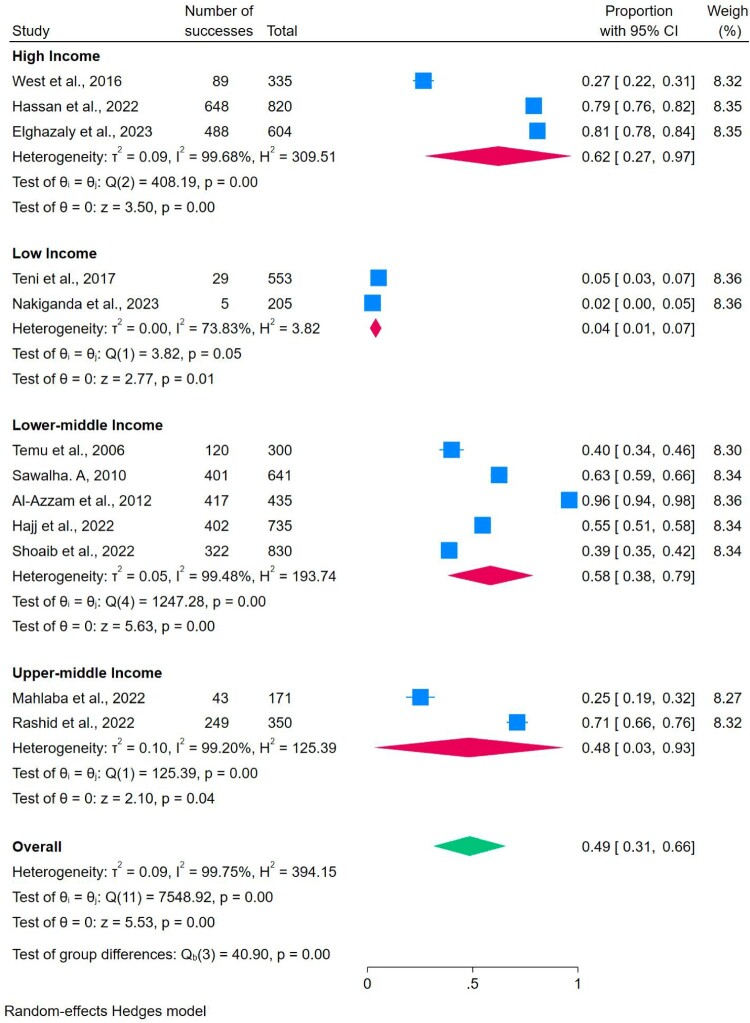
Subgroup analyses of proper storage across countries’ income levels.

The World Bank’s income classification was applied to perform the subgroup analysis in order to find the differences in proper medicine storage across countries with varying income levels. The analysis revealed that the prevalence of proper storage for general medicine was 62% in high-income countries, 48% in upper-middle-income countries, and 58% in lower-middle-income countries. Notably, low-income countries had the prevalence of only 4% for proper general medicine storage (Table 2) (Figure 4).

**Table 2.** Meta-analysis of prevalence of proper storage and disposal of medicines among countries included in the study.

Country income level	Countries included in the study	Proper storage (%)	Proper unused general medicines disposal (%)	Proper expired general medicines disposal (%)
High-income	Australia, Cyprus, Ireland, Malta, Saudi Arabia, UK, USA	62	13	10
Upper – middle-income	Brazil, China, Ecuador, Indonesia, Malaysia, Serbia, South Africa	48	8	8
Lower-middle-income	India, Iran, Jordan, Lebanon, Nepal, Nigeria, Pakistan, Tanzania, Palestine	58	17	12
Low-income	Afghanistan, Ethiopia, Liberia, Uganda	4	9	3

### Household medicine storage methods

**Figure 5.**
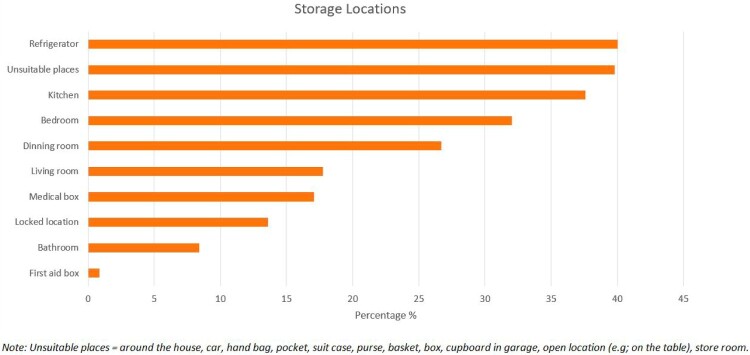
Household medicine storage methods.

Out of 61 studies, 26 studies reported the home storage methods of medicines (Figure 5). Regarding the locations, 13 studies (Abushanab et al., 2013; Al-Azzam et al., 2012; Althagafi et al., 2022; Elghazaly et al., 2023; Hajj et al., 2022; Hassan et al., 2022; Kesentseng Jackson Mahlaba et al., 2022; Rashid et al., 2022; Reddy et al., 2014; Sawalha, 2010; Shoaib et al., 2022; Sweileh et al., 2010; Teni et al., 2017; West et al., 2016) reported that the refrigerator was the most popular place where people stored medicines ranging from 5.2% in Ethiopia (Teni et al., 2017) to 94.7% in Jordan (Al-Azzam et al., 2012). Eighteen studies reported that the second most common place for storing medicines was in unsuitable places, such as cars, which typically have high temperatures, as well as in handbags, suitcases, and clothing pockets, where labels and packaging can be damaged and the medicines are easily accessible to children (Hajj et al., 2022; Hassan et al., 2022; Kesentseng Jackson Mahlaba et al., 2022; Mitiku et al., 2024; Nakiganda et al., 2023; Naser et al., 2021; Rashid et al., 2022; Temu et al., 2006; Teni et al., 2017; Terzic-Supic et al., 2019; West et al., 2016; Yimenu et al., 2020). Fourteen studies (Al-Azzam et al., 2012; Fernandes et al., 2020; Hajj et al., 2022; Hassan et al., 2022; Kesentseng Jackson Mahlaba et al., 2022; Martins et al., 2017; Mina et al., 2019; Naser et al., 2021; Rashid et al., 2022; Shoaib et al., 2022; Terzic-Supic et al., 2019; Vellinga et al., 2014; Watkins et al., 2022; West et al., 2016) reported storing medicines in the kitchen, with a range of 17% in Malaysia (Rashid et al., 2022) and 82.7% in the UK (Watkins et al., 2022).

### Determinants of proper and improper storage of medicines

Five studies reported determinants of proper storage of medicine (Al-Samydai et al., 2022; Elghazaly et al., 2023; Gregorian et al., 2020; Martins et al., 2017; Yin et al., 2019). Education (Yin et al., 2019), storage counselling (Gregorian et al., 2020), presence of chronic illness (Elghazaly et al., 2023; Gregorian et al., 2020), household size (Gregorian et al., 2020), number of children in the household (Martins et al., 2017), and medicine storage knowledge (Al-Samydai et al., 2022) had significantly positive relationships with proper medicine storage. Being male (Martins et al., 2017) and older age (≥65 years old) (Martins et al., 2017) had significantly negative relationships with proper medicine storage (Table 3).

**Table 3.** Relationship with determinants and proper storage.

Variables	Relationship with proper storage	*p*-value
Education level	Positive	0.006 ^5^
Storage counselling from healthcare provider	Positive	0.026 ^71^
Presence of chronic illness	Positive	<0.001 ^34^, 0.027 ^71^
Household size	Positive	0.00008 ^71^
Number of children in the household	Positive	<0.001 ^73^
Medicine storage knowledge	Positive	<0.001 ^30^
Age (≥65 years old)	Negative	<0.001 ^73^
Gender (male)	Negative	0.039 ^73^

### The prevalence of proper disposal

**Figure 6.**
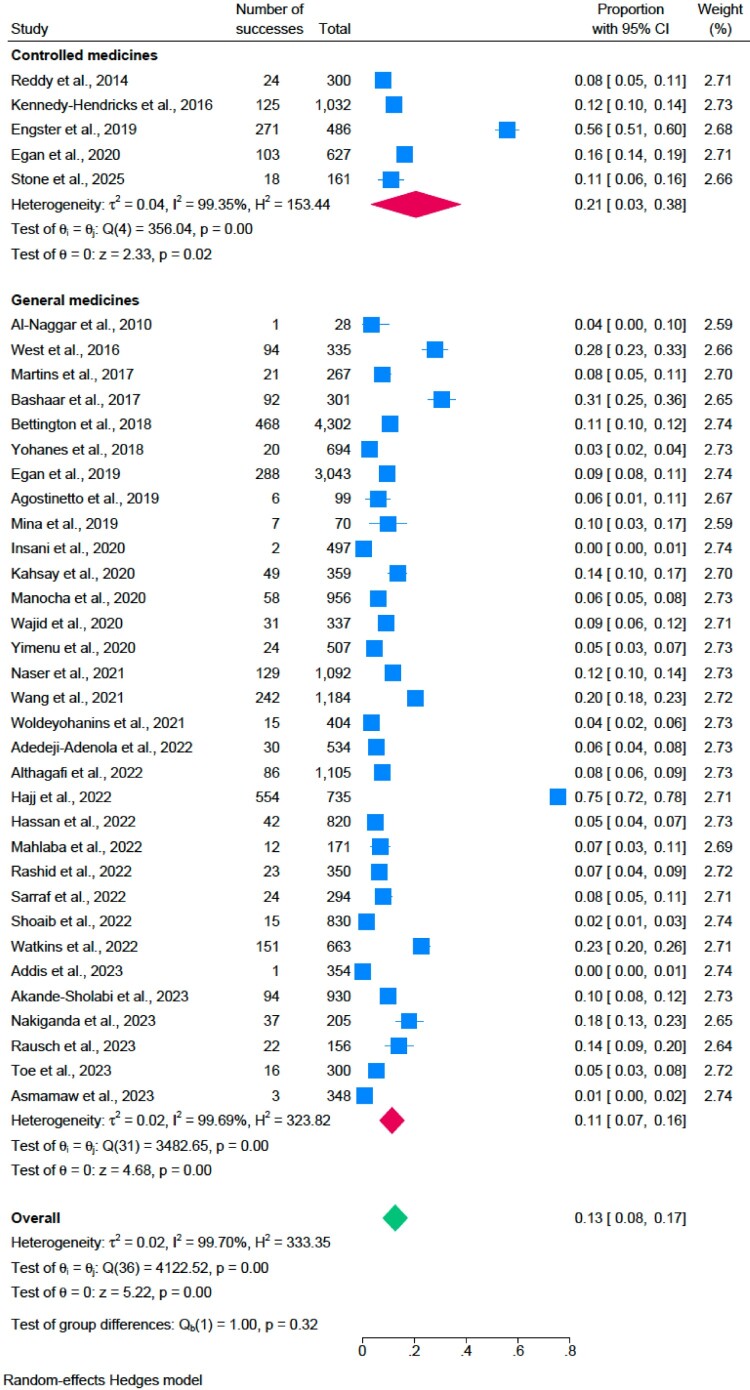
The prevalence of proper disposal of unused medicine.

**Figure 7.**
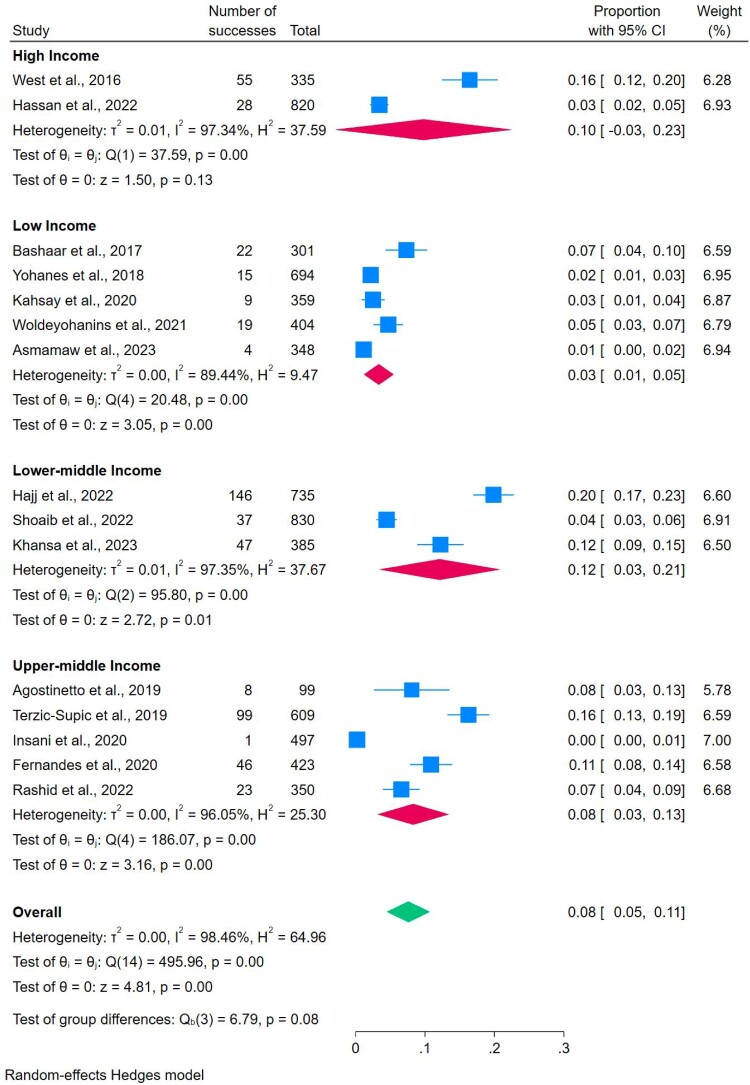
The prevalence of proper disposal of expired medicine across countries’ income levels.

Out of 61 studies, 37 studies (Addis, 2023; Adedeji-Adenola et al., 2022; Akande-Sholabi et al., 2023; Al-Naggar, 2010; Althagafi et al., 2022; Asmamaw et al., 2023; Bashaar et al., 2017; Bettington et al., 2018; Egan et al., 2019; Egan et al., 2020; Engster et al., 2019; Hajj et al., 2022; Hassan et al., 2022; Insani et al., 2020; K. J. Kahsay et al., 2020; Kennedy-Hendricks et al., 2016; Lenita et al., 2019; Mahlaba et al., 2022; Manocha et al., 2020; Martins et al., 2017; Mina et al., 2019; Nakiganda et al., 2023; Naser et al., 2021; Rashid et al., 2022; Rausch et al., 2023; Reddy et al., 2014; Sarraf et al., 2022; Shoaib et al., 2022; Stone et al., 2025; Toe et al., 2023; Wajid et al., 2020; Wang et al., 2021; Watkins et al., 2022; West et al., 2016; Woldeyohanins et al., 2021; Yimenu et al., 2020; Yohanes & Mulu, 2018) reported proper disposal of unused medicines, and 15 studies (Asmamaw et al., 2023; Bashaar et al., 2017; Fernandes et al., 2020; Hajj et al., 2022; Hassan et al., 2022; Insani et al., 2020; Kahsay et al., 2020; Khansa et al., 2023; Lenita et al., 2019; Rashid et al., 2022; Shoaib et al., 2022; Terzic-Supic et al., 2019; West et al., 2016; Woldeyohanins et al., 2021; Yohanes & Mulu, 2018) reported proper disposal of expired medicines among households. A random-effects model was applied to analyse the prevalence of unused (I^2^ **=** 99.70%; *p* = 0.00; Q = 4122.52) and expired medicine disposal (I^2^ **=** 98.46%; *p* = 0.00; Q = 495.96). The prevalence of proper disposal of unused medicine (13%; 95% CI: 0.08-0.17) was higher than expired medicine (8%; 95% CI: 0.05-0.11) (Figures 6 and 7).

#### Proper medicine disposal and countries’ income levels

**Figure 8.**
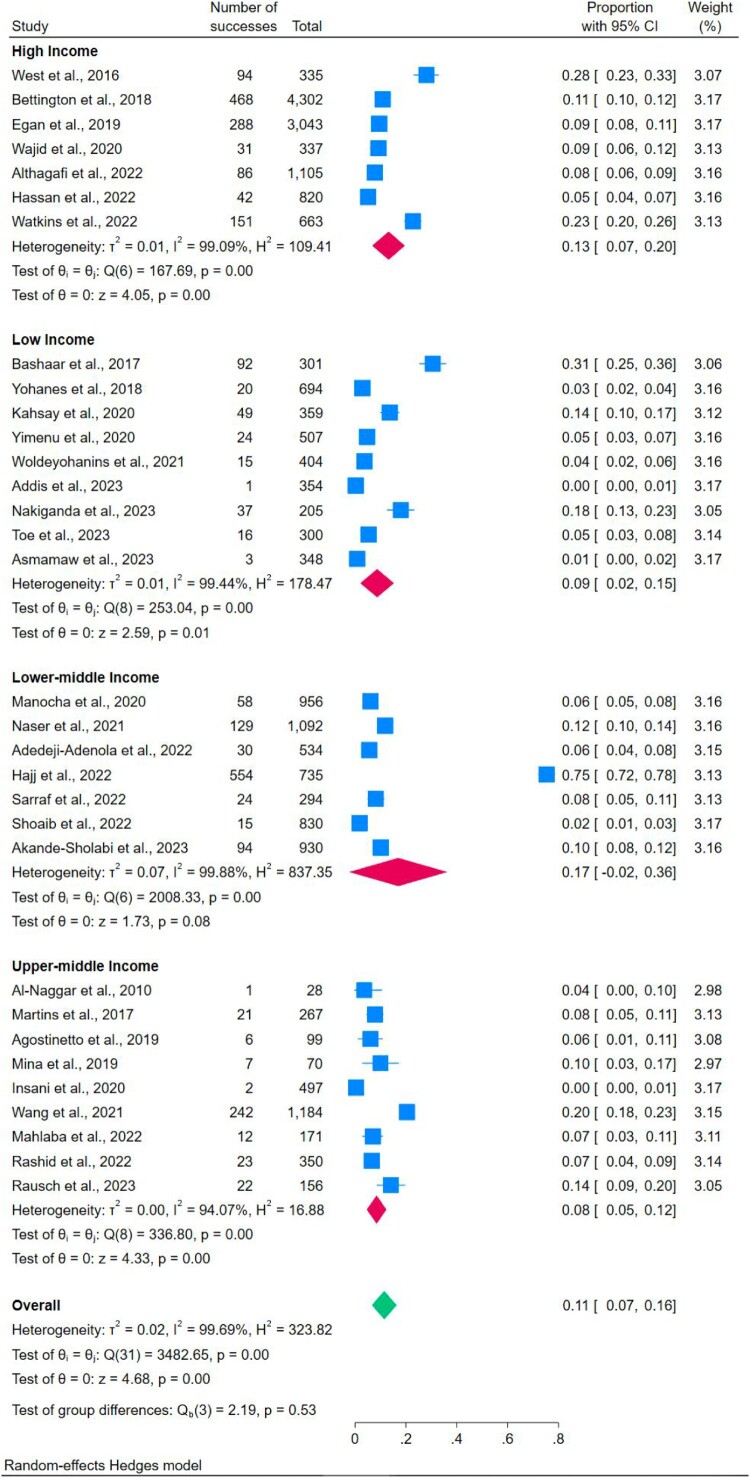
Subgroup analyses of proper disposal of unused medicine across countries’ income levels.

To assess differences in the prevalence of proper medicine disposal across income levels of countries, a subgroup analysis was performed. The analysis revealed that lower-middle-income countries had the highest prevalence of proper disposal for both unused and expired medicines (Table 2) (Figure 7 and Figure 8).

### Medicine disposal methods

**Figure 9.**
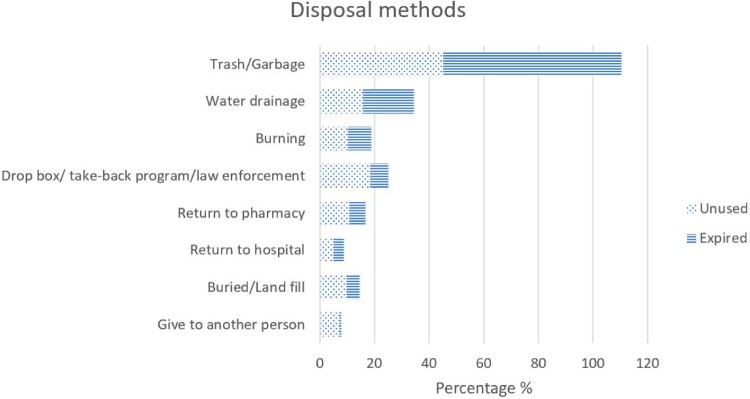
Medicine disposal methods.

Forty-six studies reported the medicine disposal methods among households (Addis, 2023; Adedeji-Adenola et al., 2022; Akande-Sholabi et al., 2023; Al-Naggar, 2010; Althagafi et al., 2022; Aluko et al., 2022; Asmamaw et al., 2023; Bashaar et al., 2017; Bettington et al., 2018; Calderón & Tarapués, 2021; Egan et al., 2019; Egan et al., 2020; Engster et al., 2019; Fernandes et al., 2020; Hajj et al., 2022; Hassan et al., 2022; Insani et al., 2020; Kahsay et al., 2020; Kennedy-Hendricks et al., 2016; Khansa et al., 2023; Lenita et al., 2019; Mahlaba et al., 2022; Manocha et al., 2020; Martins et al., 2017; Mina et al., 2019; Nakiganda et al., 2023; Naser et al., 2021; Nisha et al., 2022; Rashid et al., 2022; Rausch et al., 2023; Reddy et al., 2014; Sarraf et al., 2022; Shoaib et al., 2022; Stone et al., 2025; Terzic-Supic et al., 2019; Toe et al., 2023; Vellinga et al., 2014; Wajid et al., 2020; Wang et al., 2021; Watkins et al., 2022; West et al., 2016; WM-Bekele et al., 2023; Woldeyohanins et al., 2021; Yimenu et al., 2020; Yohanes & Mulu, 2018; Zorpas et al., 2018). Discarding medicines in household trash or garbage was reported in all studies, with the percentage ranging from 5.1% in Malta (West et al., 2016) to 97.3% in Saudi Arabia (Hassan et al., 2022). Thirty-two studies (78%) reported that participants returned unused medicines 1% (Addis, 2023; Yohanes & Mulu, 2018) to 26% (Hajj et al., 2022) and expired medicines 0.2% (Insani et al., 2020) to 19.9% (Hajj et al., 2022) to pharmacies or hospitals, although the rates were generally low (Figure 9).

### Determinants of proper disposal of medicines

Two studies reported an association between determinants and proper medicine disposal practice. Individuals with proper medicine disposal knowledge had a significantly positive association with proper disposal practice (OR = 4.09). Individuals with a higher educational level were more likely to dispose of medicine properly (OR = 1.75). The more family members are incapable of independently managing their medicine in households, the less improper medicine disposal practice (OR = 0.27). Unemployed people were more likely to dispose of medicine improperly (OR = 2.66) (Terzic-Supic et al., 2019). Another study reported that individuals with awareness of drug disposal programmes (AOR = 2.34) and peer influence (AOR = 3.06) were more likely to use the proper medicine disposal way (Egan et al., 2020).

**Figure 10.**
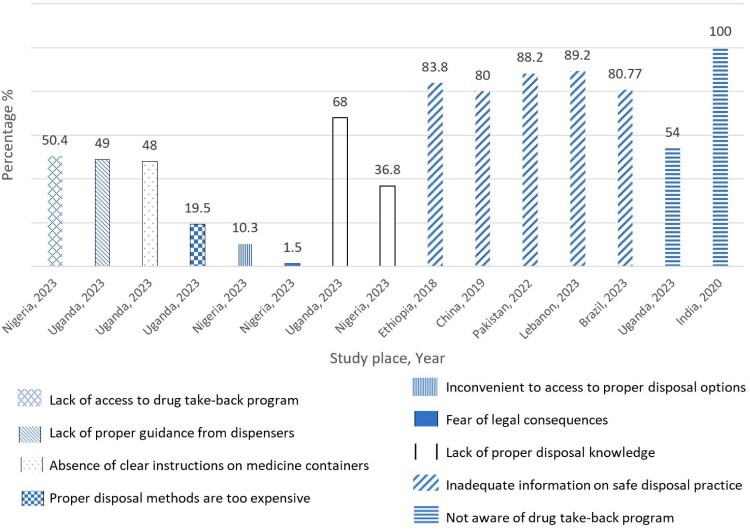
Determinants of improper disposal of medicines.

Improper disposal of medicine occurred due to unawareness of drug taking back programme (Manocha et al., 2020; Nakiganda et al., 2023), inadequate information on proper disposal practice (Khansa et al., 2023; Rausch et al., 2023; Shoaib et al., 2022; Yin et al., 2019; Yohanes & Mulu, 2018), lack of proper disposal knowledge (Akande-Sholabi et al., 2023; Nakiganda et al., 2023), fear of legal consequences (Akande-Sholabi et al., 2023), inconvenience to access to proper disposal options (Akande-Sholabi et al., 2023), expensive proper disposal methods (Nakiganda et al., 2023), absence of clear instructions on medicine containers (Nakiganda et al., 2023), lack of proper guidance from dispensers (Nakiganda et al., 2023), and lack of access to drug take-back programme (Akande-Sholabi et al., 2023). The percentage of reported determinants is shown in Figure 10.

## Discussion

### Storage

The overall prevalence rate for proper storage, according to the included studies, was 49%. Only about half of the people around the world stored the medicine properly in their households. Based on the findings, the storage locations are diverse across all geographic locations. The refrigerator was found to be the most chosen place to store medicines at home (Abushanab et al., 2013; Al-Azzam et al., 2012; Althagafi et al., 2022; Elghazaly et al., 2023; Hajj et al., 2022; Hassan et al., 2022; Kesentseng Jackson Mahlaba et al., 2022; Rashid et al., 2022; Sawalha, 2010; Shoaib et al., 2022; Sweileh et al., 2010; Teni et al., 2017). Even though certain medications, such as insulin injections and eye and ear drops, which are highly sensitive to heat, should be stored in the refrigerator to maintain their effectiveness. Some liquid medications may require refrigeration to prevent the growth of bacteria. In addition, the manufacturer's instructions on the packaging or in the patient information leaflet explicitly state to do so. This is the most reliable guide for proper storage. In Africa and Asian countries with high temperatures and humidity, refrigerators were considered a safe and proper storage location (Althagafi et al., 2022). However, this generalisation is pharmacologically inaccurate for some medicines and can be harmful in some situations. While refrigeration of heat-sensitive medications is critical, many other drugs are not designed for cold storage and may lose potency or stability when refrigerated. For example, some antibiotic suspensions, such as clarithromycin or clindamycin, should not be refrigerated because their viscosity changes can occur in the refrigerator (Abualhasan et al., 2015). The climate and temperature of each country’s location should be considered while defining the safe and proper storage location. The assumption was that keeping them in the refrigerator was appropriate because keeping the medicines at a low temperature is better than at a high temperature, especially in tropical countries. Therefore, public education campaigns, including specific instructions tailored to regional climates and specific storage requirements of medicines, are recommended.

This systematic review showed that household medicine storage was more common among people in high-income countries compared to those in low-income countries within the past decade. It indicated the economic inequality in accessing medicines. People in high-income or upper-middle-income countries could afford to purchase medication outright or indirectly access the medications through their out-of-pocket insurance. This was congruent with WHO’s statement that countries with a higher growth rate of GDP per capita were more likely to have higher health expenditures. The cost of drugs can be a substantial burden on a household's budget for people in low-income countries. Instead of buying a surplus to have on hand, people from low-income countries often purchase only what is immediately necessary for a specific course of treatment. In addition, the healthcare infrastructure in many low-income countries is often strained. This can lead to frequent drug shortages or stock-outs at public health facilities. When medicines are scarce, people may be unable to obtain them in the first place, or they may be rationed, meaning they are only given the exact dose needed for their treatment. It underlines the need for policy efforts to improve equitable accessibility of essential medicines in low-income settings through universal health coverage or programmes to reduce out-of-pocket expenditures.

According to the study from the UK, Ireland, Brazil, and Malta, most participants stored their medicines in the kitchen. The kitchen can be inappropriate to store medicines, as humidity and heat coming from cooking may change the properties of medicines and affect their potency and safety (Yin et al., 2019). Poor consumers’ knowledge about medicine storage causes improper storage of unused medicines in households (Al-Samydai et al., 2022; Martins et al., 2017). This widespread lack of knowledge about proper storage signifies a breakdown in the medicine use process, particularly in the effective communication of essential information. Dispensing policies were recommended. Pharmacists must provide clear verbal and written storage instructions with every dispensed medicine since people are less aware of the impact of improper storage, such as less effectiveness of medicine or increased side effects.

This study found that gender was strongly associated with the proper household medicine storage practice (Martins et al., 2017; Yin et al., 2019). Home medicines were generally managed by females throughout the world. Studies showed that gender roles affect improper storage of medicine when males were in charge (Martins et al., 2017), except in cultures like China, where men are educated and traditionally responsible for the household (Yin et al., 2019). However, cultural variations were present. Chinese men paid more attention to the proper storage of medicines than women (Yin et al., 2019). This might be the Chinese culture of men taking care of the family and household. Additionally, in China, men are easily accessible to higher education (Yin et al., 2019). In contrast, a Brazilian study showed that improper storage of medicines was more frequent when a male gender was the person in charge of medicines (Martins et al., 2017). These sociocultural factors should be taken into account in the interventions to promote proper storage practices.

Educational attainment was a strong determinant of proper storage and disposal of medicines, since most studies reported that a higher education level is positively associated with the amount of medicines stored and proper storage practice (Abushanab et al., 2013; Sawalha, 2010; Sweileh et al., 2010; Temu et al., 2006; Yin et al., 2019). On the other hand, those with lower education levels showed higher rates of improper practices (Martins et al., 2017; Terzic-Supic et al., 2019). People with higher educational backgrounds showed better health awareness, including good medicine home storage, disposal, and usage behaviours and compliance with the storage instructions (Yin et al., 2019; Yousif, 2002). However, as mentioned in the previous studies, educated people prefer to buy and store medicines at home for future self-therapy (Abushanab et al., 2013; Sawalha, 2010; Sweileh et al., 2010). This paradox highlights that simply having an education does not guarantee the safe use of medicine. Policy interventions must therefore go beyond general education and focus on specific health literacy regarding the proper storage of household medicines. The necessity of health education integration in school curricula to educate children and adolescents and broadening the outreach to parents and guardians through workshops, school newsletters, or informational pamphlets represents a crucial policy pathway for long-term improvements in public health outcomes.

### Disposal

The overall prevalence of proper disposal of unused and expired medicines remains critically low at 10% and 7%, respectively. Disposing of the trash or garbage was the most common method for both unused and expired medicines, which poses significant environmental and public health risks. Many participants also reported being unaware of drug take-back programmes or proper disposal methods (Althagafi et al., 2022; Rausch et al., 2023). This information indicated an urgent need for providing public disposal bins for expired medicines, enforcing clear labelling on medicine packaging with disposal instructions, enhancing public awareness campaigns focused on the environmental and health hazards of improper disposal, and finally, establishing and promoting drug take-back programmes at pharmacies and healthcare facilities. Moreover, using primary health care facilities, such as a local clinic or pharmacy, as drop-off sites is also recommended. Especially in the low-income countries, pharmacists may need to encourage the patients or consumers to use those primary health facilities as a collection point. To successfully implement this programme, financial responsibility is crucial. Therefore, pharmaceutical companies should be included as partners to share the associated costs.

Proper disposal of unused medicines among healthcare professionals (HCPs) and healthcare students revealed significant disparities across different countries. In Uganda, over three-quarters of healthcare students demonstrated good knowledge regarding proper disposal because they included course modules on medicine management, storage, and disposal, such as pharmacotherapeutics, in their curriculum (Nakiganda et al., 2023). Conversely, in Nepal (Sarraf et al., 2022) and Nigeria (Akande-Sholabi et al., 2023), more than half of HCPs exhibited poor knowledge concerning proper disposal methods. This deficiency may be due to unawareness of the guidelines and poor national and local policies on the proper disposal of unused and expired medicines. Thus, to ensure HCPs are well-informed and compliant, there is a need for enhanced knowledge to drive effective disposal behaviours. Continuous professional education can strengthen their ability to guide the public. Establishing and enforcing national and local policies that provide clear guidelines for the disposal of unused and expired medicines should also be emphasised.

Participants in lower-middle-income countries were more likely to use expired medicines. The people did not know how to dispose of expired medicines, or their country might not have a drug take-back system to return the expired medicines and medicines that can cause side effects (Adedeji-Adenola et al., 2022; Insani et al., 2020; Sarraf et al., 2022; West et al., 2016). The consequences of having these medicines at home can be risky to children, elderly people, and pets due to medical composition changes, misuse, or accidental poisoning. The development and enforcement of clear, nationwide guidelines for household medicine storage and disposal must be prioritised by local health departments or policymakers. Although the WHO has a standard guideline for drug storage and disposal, the national guideline for drug storage and disposal for households in each country should be created and fine-tuned according to the different geographical locations and environmental conditions, as well as cultural suitability and local practices. Moreover, public educational campaigns about the proper storage and disposal of home medications are also required to highlight the significant environmental and public health risks associated with improper disposal.

## Limitation

The current systematic review had certain limitations, like other studies. This might have missed some relevant articles because only English-language articles and published studies were included. The sample size variation among included studies ranged from 28 to 4,302 participants; therefore, the meta-analysis showed high heterogeneity. Subgroup analyses across countries with varying income levels were conducted to see the variation, but the number of studies in some subgroups was limited.

## Conclusion

This systematic review of 61 studies across diverse regions explored the prevalence and determinants of proper medicine storage and disposal. Findings showed that 40% of unused medicines were stored in refrigerators, while 100% of unused and expired medicines were disposed of in the household trash. The overall prevalence rate for proper storage was 45%, and proper disposal of unused and expired medicines was 13% and 8%, respectively. Proper storage was positively associated with education of people in charge of household medicines, storage counselling, presence of chronic illness, household size, number of children in the household, and medicine storage knowledge; and negatively associated with age and male gender of the household medicine in charge. Improper disposal occurred due to unawareness or lack of drug taking back programme, inadequate information on safe disposal practice, lack of proper disposal knowledge, fear of legal consequences, inconvenience, high costs, absence of clear instructions on medicine containers, and lack of proper guidance from dispensers. Proper disposal of medicine was associated with awareness, peer influence, knowledge, higher education, and having family members needing assistance with medicine management. These findings emphasise the critical need for public education activities and targeted educational interventions, including awareness campaigns, workshops, and continuing medical education for healthcare professionals, to improve proper storage and disposal practices. This review highlights the necessity of public health policies and standard protocols for proper household medicine disposal to reduce the potential risk of improper disposal.

## Authorship contribution statement

All authors (G.N.C., S.M.T., S.W., A.T., and T.K.) developed the conceptualisation of the research and design of the work. All authors discussed and developed search strategies. G.N.C. and A.T. searched and checked the databases according to the inclusion and exclusion criteria. G.N.C. and A.T. performed data extraction and discussed with T.K. and S.W. to solve the incongruency. G.N.C., S.M.T., and A.T. did the quality assessment. G.N.C., S.W., and A.T. analysed the data. S.M.T. and T.K. provided advice on meta-analysis methodology. G.N.C. wrote the draft of the paper. S.W., A.T., S.M.T., and T.K. contributed to reviewing and revising the paper. All authors read and approved the final manuscript. T.K., A.T., and S.W. are guarantors of this work and take responsibility for the data integrity and the data-analysis accuracy.

## Supplementary Material

Supplementary.docx
