# Supplementary material for: Global patterns and determinants of household medicine storage and disposal: a systematic review and meta-analysis
Source: J Pharm Policy Pract. 2026 Jan 20;19(1):2601936. doi: 10.1080/20523211.2025.2601936 (PMC12821371; doi:10.1080/20523211.2025.2601936)
Supplement: Supplementary.docx [file JPPP_A_2601936_SM5846.docx]

**Global Patterns and Determinants of Household Medicine Storage and Disposal: A Systematic Review and Meta-analysis**

Supplementary

Appendix A: Search results

| Database | Search Date | Keyword | | No. of articles found |
| --- | --- | --- | --- | --- |
| PubMed | 14.12.2023 | #1  #2  #3  #4  #5  #6  #7 | (unused) OR (expir*)  (((medicine) OR (drug)) OR (pharmaceutical)) OR (medication)  (((stor*) OR (dispos*)) OR (wast*)) OR (destroy*)  ((house*) OR (home)) OR (resident*)  (hospital) OR (pharmacy)  #1 AND #2 AND #3 AND #4 NOT #5  (((((unused) OR (expir*)) AND ((((medicine) OR (drug)) OR (pharmaceutical)) OR (medication))) AND ((((stor*) OR (dispos*)) OR (wast*)) OR (destroy*))) AND (((house*) OR (home)) OR (resident*))) NOT ((hospital) OR (pharmacy))  Filters; 1990-2023, English | 114 |
| Scopus | 14.12.2023 | (unused) OR (expir*) AND (medicine) OR (drug) OR (pharmaceutical) OR (medication) AND (stor*) OR (dispos*) OR (wast*) OR (destroy*) AND (house*) OR (home) OR (resident*) AND NOT (hospital) OR (pharmacy) in Title, Abstract, Keywords.  Filters; 1990-2023, Article types, English | | 172 |
| ScienceDirect | 14.12.2023 | ("unused medicine" OR "expired medicine") AND (store OR dispose OR waste OR destroy) AND (house OR home OR resident) NOT (hospital OR pharmacy)  Filters; research articles, 1990-2023 | | 98 |
| SpringerLink | 14.12.2023 | ("unused medicine" OR "expired medicine") AND (stor* OR dispos* OR wast* OR destroy*) AND (house* OR home OR resident*)  Filters; English, Article, 1990 - 2023 | | 224 |
| EBSCO | 14.12.2023 | ((unused) OR (expir*))  AND ((medicine) OR (drug) OR (pharmaceutical) OR (medication))  AND ((stor*) OR (dispos*) OR (wast*) OR (destroy*))  AND ((house*) OR (home) OR (resident*))  NOT ((hospital) OR (pharmacy)) in abstracts  Filters; Peer reviewed, 1990 - 2023, Academic Journals, English | | 214 |
| Total | | | | 822 |

Appendix B: Checklist of QualSyst Tool for quantitative studies

| No. | Question |
| --- | --- |
| Q1 | Question / objective sufficiently described? |
| Q2 | Study design evident and appropriate? |
| Q3 | Method of subject/comparison group selection or source of information/input variables described and appropriate? |
| Q4 | Subject (and comparison group, if applicable) characteristics sufficiently described? |
| Q5 | If interventional and random allocation was possible, was it described? |
| Q6 | If interventional and blinding of investigators was possible, was it reported? |
| Q7 | If interventional and blinding of subjects was possible, was it reported? |
| Q8 | Outcome and (if applicable) exposure measure(s) well defined and robust to measurement / misclassification bias? Means of assessment reported? |
| Q9 | Sample size appropriate? |
| Q10 | Analytic methods described/justified and appropriate? |
| Q11 | Some estimate of variance is reported for the main results? |
| Q12 | Controlled for confounding? |
| Q13 | Results reported in sufficient detail? |
| Q14 | Conclusions supported by the results? |

Appendix C: Checklist of QualSyst Tool for quanlitative studies

| No. | Question |
| --- | --- |
| Q1 | Question / objective sufficiently described? |
| Q2 | Design evident and appropriate to answer study question? (If the study question is not clearly identifi ed, infer appropriateness from results/conclusions.) |
| Q3 | Context for the study clear? |
| Q4 | Connection to a theoretical framework / wider body of knowledge? |
| Q5 | Sampling strategy described, relevant and justified? |
| Q6 | Data collection methods clearly described and systematic? |
| Q7 | Data analysis clearly described and systematic.? |
| Q8 | Use of verification procedure(s) to establish credibility? |
| Q9 | Conclusions supported by the results? |
| Q10 | Reflexivity of the account? |

Appendix D: Quality assessment scores of quantitative studies

| Author (year) | Average score given by three researchers for each question of the tool used in the current systematic review | | | | | | | | | | | | | | Total average |
| --- | --- | --- | --- | --- | --- | --- | --- | --- | --- | --- | --- | --- | --- | --- | --- |
|  | Q 1 | Q 2 | Q 3 | Q 4 | Q 5 | Q 6 | Q 7 | Q 8 | Q9 | Q10 | Q11 | Q12 | Q13 | Q14 |  |
| Zargarzadeh et al., 2005 | 2 | 2 | 2 | 2 | N/A | N/A | N/A | 2 | 2 | 2 | 2 | N/A | 2 | 2 | 1 |
| Temu et al., 2006 | 2 | 2 | 2 | 1 | N/A | N/A | N/A | 0 | 2 | 2 | 0 | N/A | 2 | 2 | 0.75 |
| Sawalha. A, 2010 | 2 | 2 | 2 | 2 | N/A | N/A | N/A | 2 | 1 | 2 | 1 | N/A | 2 | 2 | 0.9 |
| Sweileh et al., 2010 | 2 | 2 | 2 | 2 | N/A | N/A | N/A | 2 | 1 | 2 | 2 | N/A | 2 | 0 | 0.85 |
| Al-Azzam et al., 2012 | 1 | 2 | 2 | 2 | N/A | N/A | N/A | 1 | 2 | 2 | 2 | N/A | 1 | 1 | 0.8 |
| Abushanab et al., 2013 | 2 | 2 | 2 | 2 | N/A | N/A | N/A | 1 | 2 | 2 | 2 | N/A | 2 | 1 | 0.9 |
| Reddy et al., 2014 | 1 | 2 | 2 | 2 | N/A | N/A | N/A | 1 | 1 | 2 | 2 | N/A | 2 | 2 | 0.85 |
| Vellinga et al., 2014 | 2 | 2 | 2 | 0 | N/A | N/A | N/A | 2 | 1 | 2 | 0 | N/A | 2 | 2 | 0.75 |
| West et al., 2016 | 1 | 2 | 2 | 2 | N/A | N/A | N/A | 0 | 2 | 2 | 0 | N/A | 2 | 2 | 0.75 |
| Martins et al., 2017 | 2 | 2 | 2 | 1 | N/A | N/A | N/A | 0 | 1 | 2 | 2 | N/A | 2 | 2 | 0.8 |
| Bashaar et al., 2017 | 1 | 2 | 2 | 2 | N/A | N/A | N/A | 1 | 1 | 2 | N/A | N/A | 2 | 2 | 0.83 |
| Teni et al., 2017 | 2 | 2 | 2 | 2 | N/A | N/A | N/A | 1 | 2 | 2 | 2 | N/A | 2 | 2 | 0.95 |
| Bettington et al., 2018 | 1 | 2 | 1 | 2 | N/A | N/A | N/A | 1 | 2 | 2 | 2 | N/A | 2 | 2 | 0.85 |
| Yohanes et al., 2018 | 1 | 2 | 2 | 2 | N/A | N/A | N/A | 2 | 2 | 1 | 0 | N/A | 2 | 2 | 0.8 |
| Zorpas et al., 2018 | 2 | 2 | 2 | 1 | N/A | N/A | N/A | 1 | 1 | 1 | 1 | N/A | 2 | 2 | 0.75 |
| Egan et al., 2019 | 2 | 2 | 2 | 2 | N/A | N/A | N/A | 2 | 2 | 2 | 2 | N/A | 2 | 2 | 1 |
| Agostinetto et al., 2019 | 2 | 2 | 2 | 2 | N/A | N/A | N/A | 1 | 1 | 1 | 2 | N/A | 2 | 2 | 0.85 |
| Logesh et al., 2019 | 1 | 2 | 2 | 2 | N/A | N/A | N/A | 2 | 2 | 2 | 2 | N/A | 2 | 2 | 0.95 |
| Mina et al., 2019 | 1 | 2 | 2 | 0 | N/A | N/A | N/A | 0 | 2 | 2 | 2 | N/A | 2 | 2 | 0.75 |
| Terzic-Supic et al., 2019 | 2 | 2 | 2 | 2 | N/A | N/A | N/A | 2 | 2 | 2 | 2 | N/A | 2 | 2 | 1 |
| Huang et al., 2019 | 1 | 2 | 2 | 2 | N/A | N/A | N/A | 2 | 2 | 2 | 2 | N/A | 2 | 2 | 0.95 |
| Gregorian et al., 2020 | 2 | 2 | 2 | 2 | N/A | N/A | N/A | 0 | 2 | 2 | 2 | N/A | 2 | 2 | 0.9 |
| Insani et al., 2020 | 1 | 2 | 2 | 2 | N/A | N/A | N/A | 2 | 2 | 1 | 2 | N/A | 2 | 1 | 0.85 |
| Kahsay et al., 2020 | 1 | 2 | 2 | 2 | N/A | N/A | N/A | 2 | 2 | 2 | 2 | N/A | 2 | 2 | 0.95 |
| Manocha et al., 2020 | 1 | 2 | 2 | 1 | N/A | N/A | N/A | 2 | 2 | 1 | 2 | N/A | 2 | 2 | 0.85 |
| Fernandes et al., 2020 | 2 | 2 | 2 | 0 | N/A | N/A | N/A | 1 | 2 | 1 | 2 | N/A | 2 | 2 | 0.8 |
| Wajid et al., 2020 | 1 | 2 | 2 | 2 | N/A | N/A | N/A | 1 | 2 | 1 | 2 | N/A | 2 | 2 | 0.85 |
| Yimenu et al., 2020 | 1 | 2 | 2 | 2 | N/A | N/A | N/A | 0 | 2 | 2 | 2 | N/A | 2 | 2 | 0.85 |
| Calderón et al., 2021 | 2 | 2 | 2 | 2 | N/A | N/A | N/A | 1 | 2 | 1 | 2 | N/A | 2 | 2 | 0.8 |
| Naser et al., 2021 | 1 | 2 | 2 | 2 | N/A | N/A | N/A | 2 | 1 | 1 | 2 | N/A | 2 | 2 | 0.85 |
| Wang et al., 2021 | 1 | 2 | 2 | 2 | N/A | N/A | N/A | 2 | 2 | 2 | 2 | N/A | 2 | 2 | 0.95 |
| Woldeyohanins et al., 2021 | 1 | 2 | 2 | 2 | N/A | N/A | N/A | 2 | 2 | 1 | 2 | N/A | 2 | 2 | 0.9 |
| Adedeji-Adenola et al., 2022 | 2 | 2 | 2 | 2 | N/A | N/A | N/A | 1 | 2 | 2 | 2 | N/A | 2 | 2 | 0.95 |
| Al-Samydai et al., 2022 | 1 | 2 | 2 | 2 | N/A | N/A | N/A | 0 | 2 | 2 | 2 | N/A | 2 | 2 | 0.85 |
| Althagafi et al., 2022 | 1 | 2 | 2 | 2 | N/A | N/A | N/A | 2 | 2 | 0 | 2 | N/A | 2 | 2 | 0.85 |
| Aluko et al., 2022 | 0 | 2 | 2 | 2 | N/A | N/A | N/A | 2 | 2 | 2 | 2 | N/A | 2 | 2 | 0.9 |
| Hajj et al., 2022 | 1 | 2 | 2 | 2 | N/A | N/A | N/A | 2 | 2 | 2 | 2 | N/A | 2 | 2 | 0.95 |
| Hassan et al., 2022 | 1 | 2 | 2 | 2 | N/A | N/A | N/A | 2 | 2 | 2 | 2 | N/A | 2 | 2 | 0.95 |
| Mahlaba et al., 2022 | 2 | 2 | 2 | 2 | N/A | N/A | N/A | 2 | 1 | 0 | 2 | N/A | 2 | 2 | 0.85 |
| Jha et al., 2022 | 1 | 2 | 2 | 2 | N/A | N/A | N/A | 2 | 2 | 2 | N/A | N/A | 2 | 2 | 0.94 |
| Rashid et al., 2022 | 1 | 2 | 2 | 2 | N/A | N/A | N/A | 2 | 1 | 2 | N/A | N/A | 2 | 2 | 0.88 |
| Sarraf et al., 2022 | 1 | 2 | 2 | 2 | N/A | N/A | N/A | 2 | 2 | 2 | 2 | N/A | 2 | 2 | 0.95 |
| Shoaib et al., 2022 | 1 | 2 | 2 | 2 | N/A | N/A | N/A | 2 | 2 | 2 | 2 | N/A | 2 | 2 | 0.95 |
| Watkins et al., 2022 | 2 | 2 | 1 | 1 | N/A | N/A | N/A | 2 | 1 | 2 | 2 | N/A | 2 | 2 | 0.85 |
| Addis et al., 2023 | 1 | 2 | 2 | 1 | N/A | N/A | N/A | 1 | 2 | 2 | 1 | N/A | 2 | 2 | 0.8 |
| Akande-Sholabi et al., 2023 | 1 | 2 | 2 | 2 | N/A | N/A | N/A | 2 | 2 | 2 | 2 | N/A | 2 | 2 | 0.95 |
| Hidayati et al., 2023 | 1 | 2 | 2 | 2 | N/A | N/A | N/A | 2 | 2 | 2 | 2 | N/A | 2 | 2 | 0.95 |
| Elghazaly et al., 2023 | 1 | 2 | 2 | 2 | N/A | N/A | N/A | 2 | 2 | 2 | 1 | N/A | 2 | 2 | 0.9 |
| Khansa et al., 2023 | 1 | 2 | 2 | 2 | N/A | N/A | N/A | 2 | 2 | 2 | 2 | N/A | 2 | 2 | 0.95 |
| Nakiganda et al., 2023 | 1 | 2 | 2 | 2 | N/A | N/A | N/A | 2 | 2 | 2 | 2 | N/A | 2 | 2 | 0.95 |
| Rausch et al., 2023 | 2 | 2 | 2 | 2 | N/A | N/A | N/A | 2 | 1 | 2 | 2 | N/A | 2 | 2 | 0.95 |
| Toe et al., 2023 | 1 | 2 | 2 | 2 | N/A | N/A | N/A | 2 | 1 | 0 | 1 | N/A | 2 | 2 | 0.75 |
| Bekele et al., 2023 | 1 | 2 | 2 | 2 | N/A | N/A | N/A | 2 | 2 | 2 | 1 | N/A | 2 | 2 | 0.9 |
| Asmamaw et al., 2023 | 1 | 2 | 2 | 1 | N/A | N/A | N/A | 1 | 2 | 2 | 1 | N/A | 2 | 2 | 0.8 |
| Engster et al., 2019 | 1 | 2 | 2 | 2 | N/A | N/A | N/A | 2 | 1 | 0 | 1 | N/A | 2 | 2 | 0.75 |
| Egan et al., 2020 | 1 | 2 | 2 | 2 | N/A | N/A | N/A | 0 | 2 | 2 | 2 | N/A | 2 | 2 | 0.85 |
| Mitiku et al., 2024 | 1 | 2 | 2 | 2 | N/A | N/A | N/A | 2 | 2 | 0 | 2 | N/A | 2 | 2 | 0.85 |
| Kennedy-Hendricks et al., 2016 | 2 | 2 | 2 | 2 | N/A | N/A | N/A | 1 | 2 | 1 | 2 | N/A | 2 | 2 | 0.8 |
| Stone et al.,2024 | 1 | 2 | 2 | 1 | N/A | N/A | N/A | 1 | 2 | 2 | 1 | N/A | 2 | 2 | 0.8 |

Appendix E: Quality assessment scores of qualitative studies

| Author (year) | Average score given by three researchers for each question of the tool used in the current systematic review | | | | | | | | | | Total average |
| --- | --- | --- | --- | --- | --- | --- | --- | --- | --- | --- | --- |
|  | Q 1 | Q 2 | Q 3 | Q 4 | Q 5 | Q 6 | Q 7 | Q8 | Q9 | Q10 |  |
| Al-Naggar et al., 2010 | 2 | 2 | 2 | 2 | 2 | 2 | 1 | 2 | 2 | 2 | 0.95 |
| Kelly et al., 2018 | 1 | 2 | 2 | 2 | 1 | 2 | 2 | 2 | 2 | 2 | 0.9 |
| Bekele et al., 2023 | 1 | 2 | 2 | 2 | 2 | 1 | 1 | 2 | 1 | 2 | 0.8 |

Appendix F: Information of excluded studies

| No. | Author, Year | Title | Reasons of exclusion |
| --- | --- | --- | --- |
| 1 | AlMutairi et al., 2022 | Household medicine disposal and waste management in Ireland,  the need for a systems‑based approach | Editorial |
| 2 | Proskurova et al., 2019 | Analysis of handling practice with unused medicines in home first aid kits of the Ukrainian households | Pilot Study |
| 3 | Rogowska et al., 2019 | Pharmaceutical Household Waste Practices: Preliminary Findings from a Case Study in Poland | Preliminary finding |
| 4 | Azad et al., 2012 | Disposal practice for unused medications among the students of the international islamic university Malaysia | Low quality assessment score |
| 5 | Banwat et al., 2016 | Assessment of the storage and disposal of medicines in some homes in Jos north local government area of Plateau State, Nigeria | Low quality assessment score |
| 6 | Maeng et al., 2016 | Unused medications and disposal patterns at home: Findings from a Medicare patient survey and claims data | Low quality assessment score |
| 7 | Silvestre et al., 2017 | Frequency of unsafe storage, use, and disposal practices of opioids among cancer patients presenting to the emergency department | Low quality assessment score |
| 8 | Akici et al., 2018 | Assessment of the association between drug disposal practices and drug use and storage behaviors | Low quality assessment score |
| 9 | Ariffin et al., 2019 | Household Pharmaceutical Waste Disposal in Selangor, Malaysia—Policy, Public Perception, and Current Practices | Low quality assessment score |
| 10 | Sharma et al., 2021 | Disposal of unused antibiotics as household waste: A social driver of antimicrobial resistance | Low quality assessment score |
| 11 | Alshehri et al., 2022 | Increasing Awareness of Proper Disposal of Unused and Expired Medication Using a Knowledge-Based Disposal Management System | Low quality assessment score |
| 12 | Zalpuri et al., 2022 | Disposal practices and awareness of medicine waste management among general population of Delhi-National Capital Region, India | Low quality assessment score |
